# Visual analysis of the research hotspots in neoadjuvant therapy for patients with gastric cancer

**DOI:** 10.3389/fgstr.2023.1148787

**Published:** 2023-04-28

**Authors:** Tianxing Chen, Yating Liu, Jing Gao, Dekui Zhang

**Affiliations:** ^1^ Lanzhou University Second School of Clinical Medicine, Lanzhou, Gansu, China; ^2^ Department of Gastroenterology, Lanzhou University Second Hospital, Lanzhou, Gansu, China

**Keywords:** gastric cancer, neoadjuvant chemotherapy, research hotspot frontier, visual analysis, bibliometric study

## Abstract

**Objective:**

This study aimed to analyze the research hotspots and frontiers in the field of neoadjuvant therapy for patients with gastric cancer (GC) using bibliometric and identify its developmental trends.

**Methods:**

The literature related to neoadjuvant therapy for GC systematically retrieved between 1991 and 2021. Bibliometric methods were used to analysis the research hotspots and trends by CiteSpace and VOS-viewer software.

**Results:**

The number of studies related to neoadjuvant therapies for GC showed an upward trend. Moreover, the current research directions were mostly focused on the clinical trials and applications of neoadjuvant therapies for GC. The frontier research directions included microsatellite instability, peritoneal metastasis, randomized controlled trials, multicenter studies, and regression analysis.

**Conclusions:**

The interest and attention of researchers in this field are still growing. In the past five years, the literature related to neoadjuvant therapy and immunotherapy for gastric cancer has exploded. It is still an important period and a key stage for the development of neoadjuvant therapy for gastric cancer.

Gastric cancer (GC) is the most common malignancy of the digestive system. According to the latest data from the International Agency for Research on Cancer (IARC) GLOBOCAN 2020 (1), the morbidity and mortality rates of GC ranked fifth and fourth, respectively, among 36 cancer types in 185 countries and regions worldwide with about one million new cases and 768,000 deaths. China ranked first with a high morbidity of GC, accounting for half of the incidence and mortality rates of the total GC cases worldwide (2). The result of national cancer clinical research center in 2020 (3, 4) showed that the morbidity and mortality rates of GC were the third highest rates among malignant tumors in China. There are about 479,000 new GC cases, and 374,000 deaths. These observations showed that the research, diagnosis, and treatment of GC in China have a long way to go. Currently, the individualized comprehensive treatment strategies for GC include surgery, chemotherapy, radiotherapy, targeted drugs, and immunotherapy; however, the overall results are not effective and have a poor outcome (5, 6). In order to improve the surgical resection and proportion of radical resection rates, and reduce the risk of recurrence and metastasis of GC, neoadjuvant therapy is emerging and has shown significant benefits (7–9). Globally, the neoadjuvant therapy of GC has received more and more attention, and there are more and more related studies. Although the continuous improvement in modern medical theory and experimental knowledge is continuously developing research hotspots in the field of neoadjuvant therapy for GC, the research on, on comprehensively related research and exploration of research hotspots in neoadjuvant therapy is still very limited, and there are still many controversies. Therefore, this study aims to clarify the frontiers and development trends in this field by describing the knowledge base and current research o, and to analysis the cutting-edge and developmental trends, and applying the software to process and draw a visual view. This study can provide a reference for clinical studies of neoadjuvant therapy for gastric cancer.

## 1 Methods

### 1.1 Literature search strategy

Web of Science Core Collection (WoSCC) database from Clarivate Analytics is one of the best choices for bibliometric analysis in academia (10, 11). In this study, the WoSCC database was selected as the data source. The studies related to the new adjuvant treatment of GC from January 1, 1980, to December 31, 2021, were searched in the SCI-E database. In order to ensure the accuracy of search results, the medical subject headlines (MeSH) terms were used to identify the relevant studies (12).

### 1.2 Inclusion and exclusion criteria

**Figure 1:**
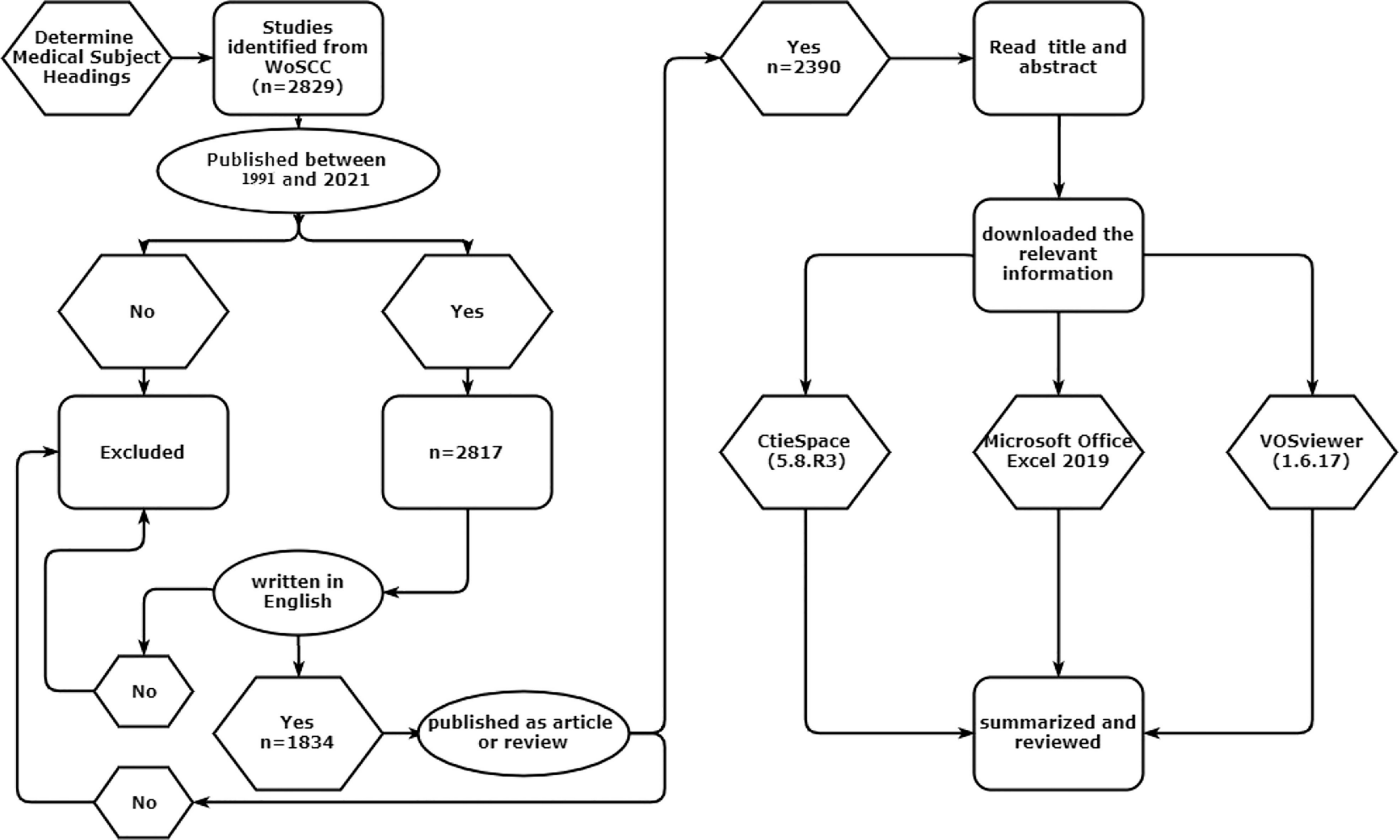
Literature screening and analysis process.

The following inclusion and exclusion criteria were adopted during this study: (1) the type of literature was limited to “article” or “review”; (2) the language was limited to English; (3) the publication period was limited from inception, to June 31, 2022. The studies that did not meet these criteria were excluded by reading its abstract. Finally, a total of 2390 articles after removing duplicated articles were included, which involve 1937 original research and 403 reviews, as shown in **Figure 1**.

### 1.3 Data processing and statistical analysis

All relevant literature were exported as “full records and cited references” and stored as “pure text”. The information of studies was imported to CiteSpace (5.8.R3) (13) and VOSviewer (14). Since the first study, was published in 1991 in the WoSCC database, duration for literature retrieval was 1991-2021. Use Microsoft Office Excel 2019 to conduct statistical analysis on the annual publication volume of literature, and fit the publication volume curve. The threshold value of each time slice was set as Top N = 50 or k = 25. The critical path algorithm is used for the cluster analysis, presentation, cooperation network of countries, research institutions, and scholars. The co-citation analysis of the journal information in the cited literature is carried using the matrix operation, and the co-citation map was drawn. Using the weighted calculation of co-word analysis, the keywords are subjected to co-occurrence cluster analysis; this results in obtaining the top 50 keywords with the highest reference surge index. Thus, the timeline view was drawn.

## 2 Result

### 2.1 Publication trend of literature

The concept of neoadjuvant chemotherapy was first proposed in a study published by Frey et al. in 1982 (15). Until 1991, published studies on neoadjuvant chemotherapy for GC were based on methotrexate and 5-fluorouracil (16). Most of the current consensus or guideline recommendations for neoadjuvant chemotherapy for gastric cancer are based on important evidence from the UK’s MAGIC (ECF chemotherapy regimen)and France’s FNCLCC/FFCD(CF chemotherapy regimen)studies (17). South Korea’s PRODGY study and China’s RESOLVE study were released at the ESMO(European Society for Medical Oncology)Annual Meeting in 2019. The PRODIGY study showed that for locally advanced gastric cancer, DOS regimen (docetaxel + oxaliplatin + Tiggio) significantly improved 3-year progression-free survival (18). RESOLVE study results showed that in the treatment of locally advanced gastric cancer, the perioperative SOX regimen significantly improved 3-year disease-free survival compared with postoperative XELOX regimen, and SOX regimen was no worse than XELOX regimen. Based on the above research results, DOS and SOX protocols can also be recommended as new adjuvant chemotherapy protocols for gastric cancer, and the above protocols have been included in perioperative chemotherapy in the 2020 edition of CSCO(Chinese Society of Clinical Oncology)guidelines for Gastric cancer. In 2009, the German POET trial showed that neoadjuvant chemoradiotherapy significantly improved pCR in patients with gastric cancer compared with neoadjuvant chemoradiotherapy (15.6% *vs*. 2.0%, P=0.03) (19). Many countries have also conducted a large number of studies on neoadjuvant therapy with targeted chemotherapy drugs. The results of the HER-FLOT study in Germany showed that FLOT chemotherapy regimen combined with trastuzua monoclonal antibody 4 cycles before surgery could enable 21.4% of AEG patients to achieve pCR and 92.9% to obtain R0 resection (20). The NEOHX study in Spain also suggested that neoadjuvant chemotherapy with XELOX protocol combined with trastuzua monoclonal antibody could achieve pCR in 8% of patients (21). In 2020, PETRARCA research results: FLOT+ human epidermal growth factor receptor 2 double target drugs (trastuzol monoclonal antibody and Pertuzol monoclonal antibody) group can effectively improve pCR and lymph node negative conversion rate. During the selected 30-year period (1991-2021), a total of 2390 published articles were obtained from the WoSCC database. From 1991 to 1994, the number of published articles was relatively small, with an average of only 3.5 articles per year. Since 2013, more than 100 articles have been published, and the fluctuations continue to increase. The average number of published articles in 2017-2021 is about 247.2. The number of published articles in the past five years accounted for 51.72% of the total number of published articles. The number of published articles will reach a peak of 314 in 2021.

**Figure 2:**
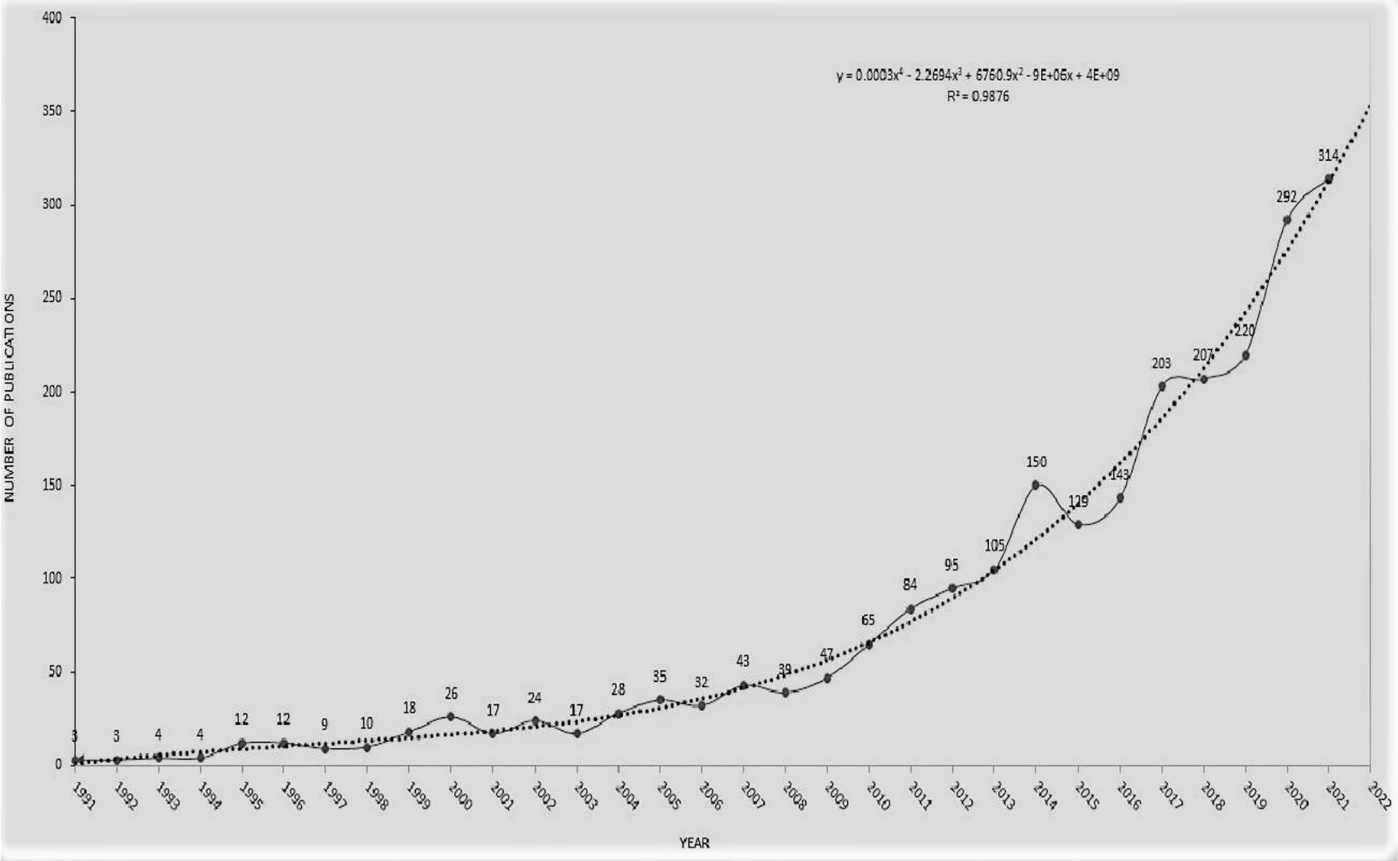
Publication trends of literature on GC neoadjuvant chemotherapy from 1991 to 2021.

Based on these founds, the published articles in the field of GC neoadjuvant therapy in the past 30 years showed an exponential trend (R2 = 0.9876) (**Figure 2**). According to the results of the fitting curve, the number of studies on neoadjuvant therapy for gastric cancer may reach nearly 350 in 2022. These results suggest that neoadjuvant therapy of gastric cancer has been paid more and more attention in the treatment of gastric cancer.

### 2.2 Distribution of literature sources based on countries and institutions

**Figure 3:**
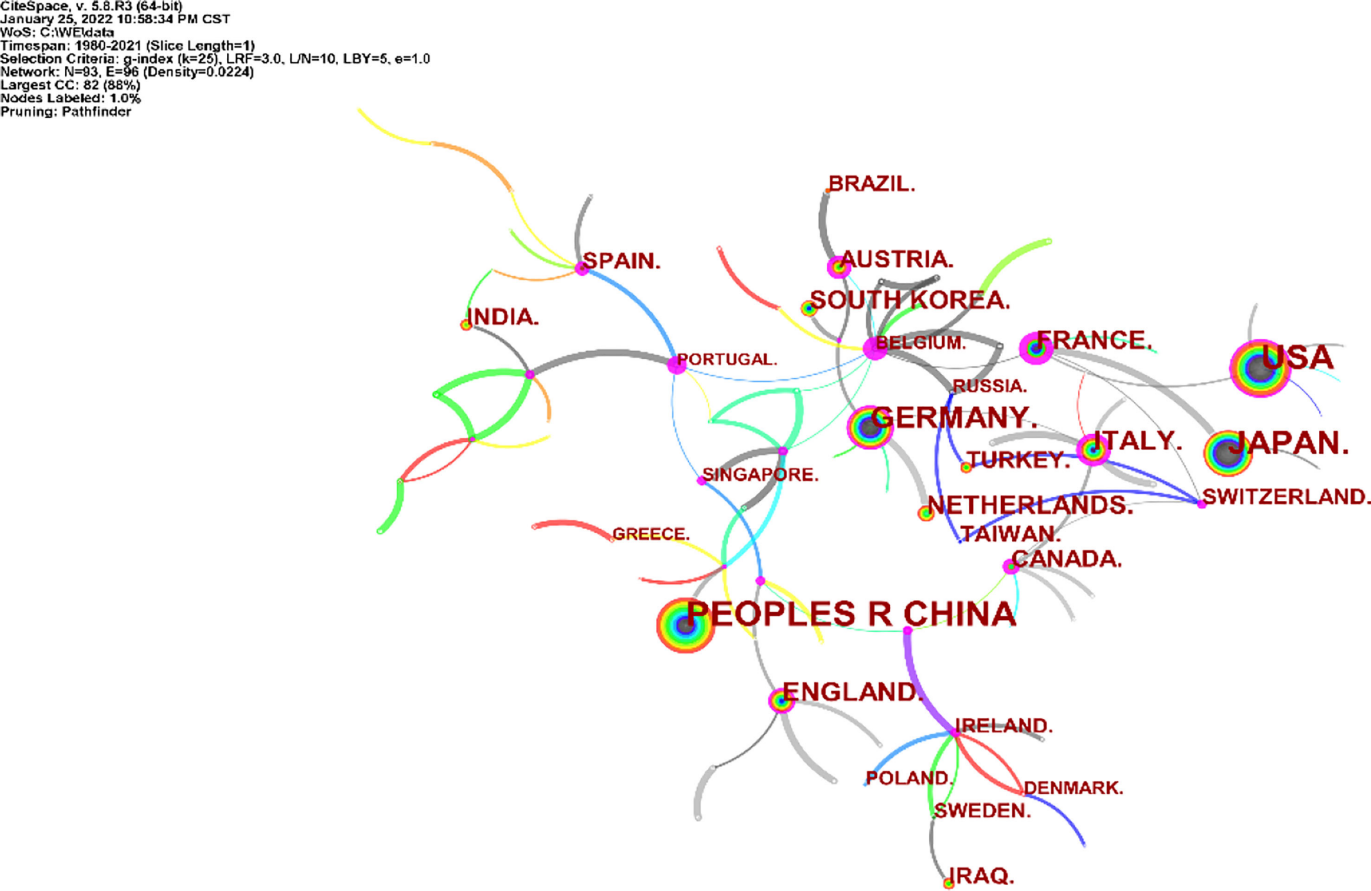
Distribution of literature sources related to neoadjuvant chemotherapy for GC from 1991 to 2021 based on countries.

A total of 2,390 articles on neoadjuvant therapy for gastric cancer were published in 93 countries and regions. Among these countries and regions, China, the United States and Japan rank among the top three countries with the largest number of articles. China has the largest number of published articles (n=488), accounting for 20.42% of the total number of published articles. The United States ranks in the middle (betweenness centrality = 0.52), as shown in **Figure 3**.

**Figure 4:**
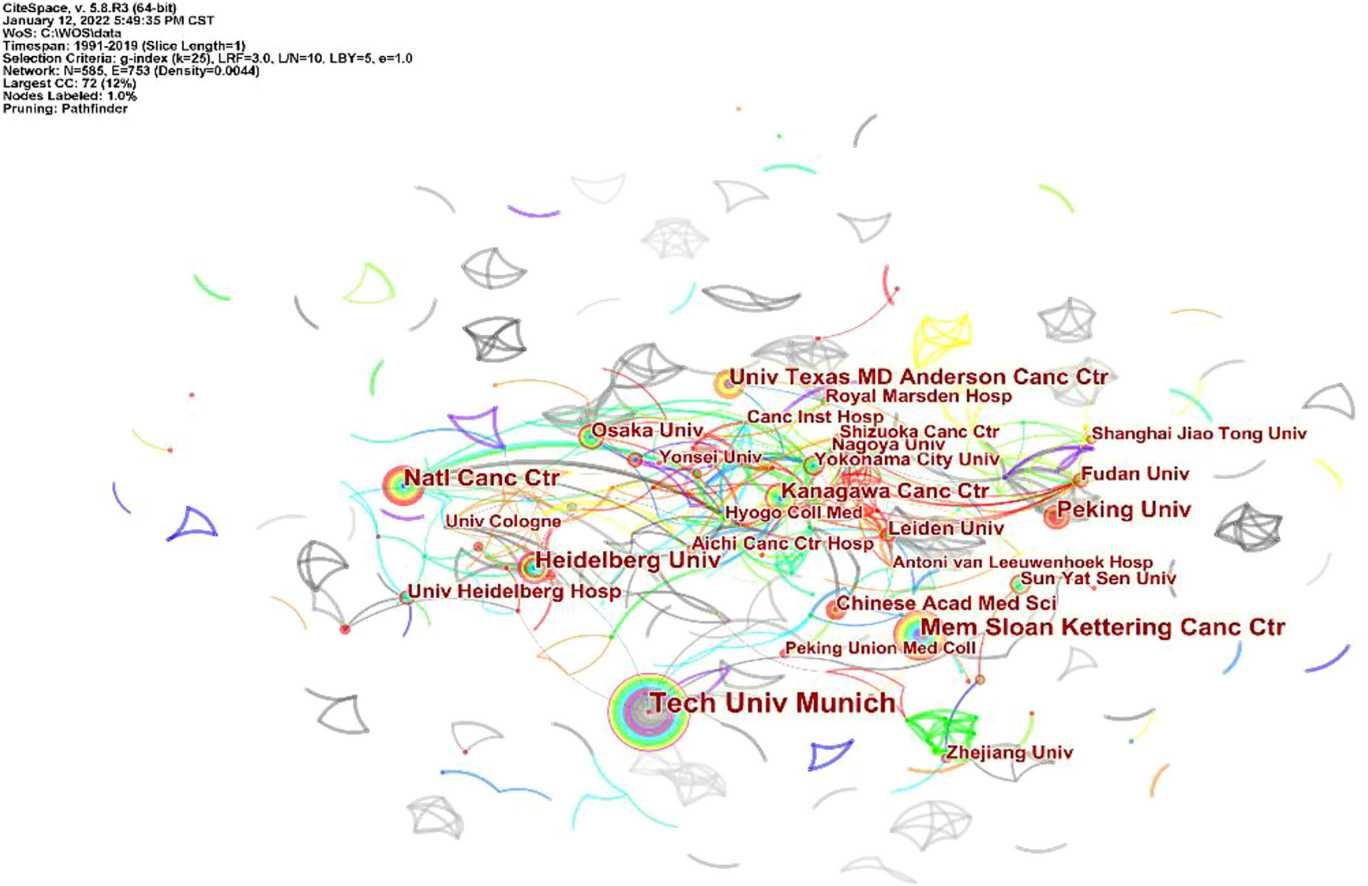
Distribution of literature sources related to neoadjuvant chemotherapy for GC from 1991 to 2021 based on institutions.

A total of 555 institutions participated in the publication of 2,390 articles on GC neoadjuvant therapy, of which the top 10 institutions published 341 articles, accounting for 14.27% of all articles. Germany’s Technical University of Munich published 77 articles, the highest number and betweenness centrality (betweenness centrality = 0.11). Among the top 10 institutions are the Sloan Kettering Cancer Center in the US, the National Institute of Cancer Center in Japan, Peking University in China (ranked fifth) and the Chinese Academy of Medical Sciences (ranked tenth) (**Figure 4**).

### 2.3 Distribution and co-citation of journal sources and authors

**Figure 5:**
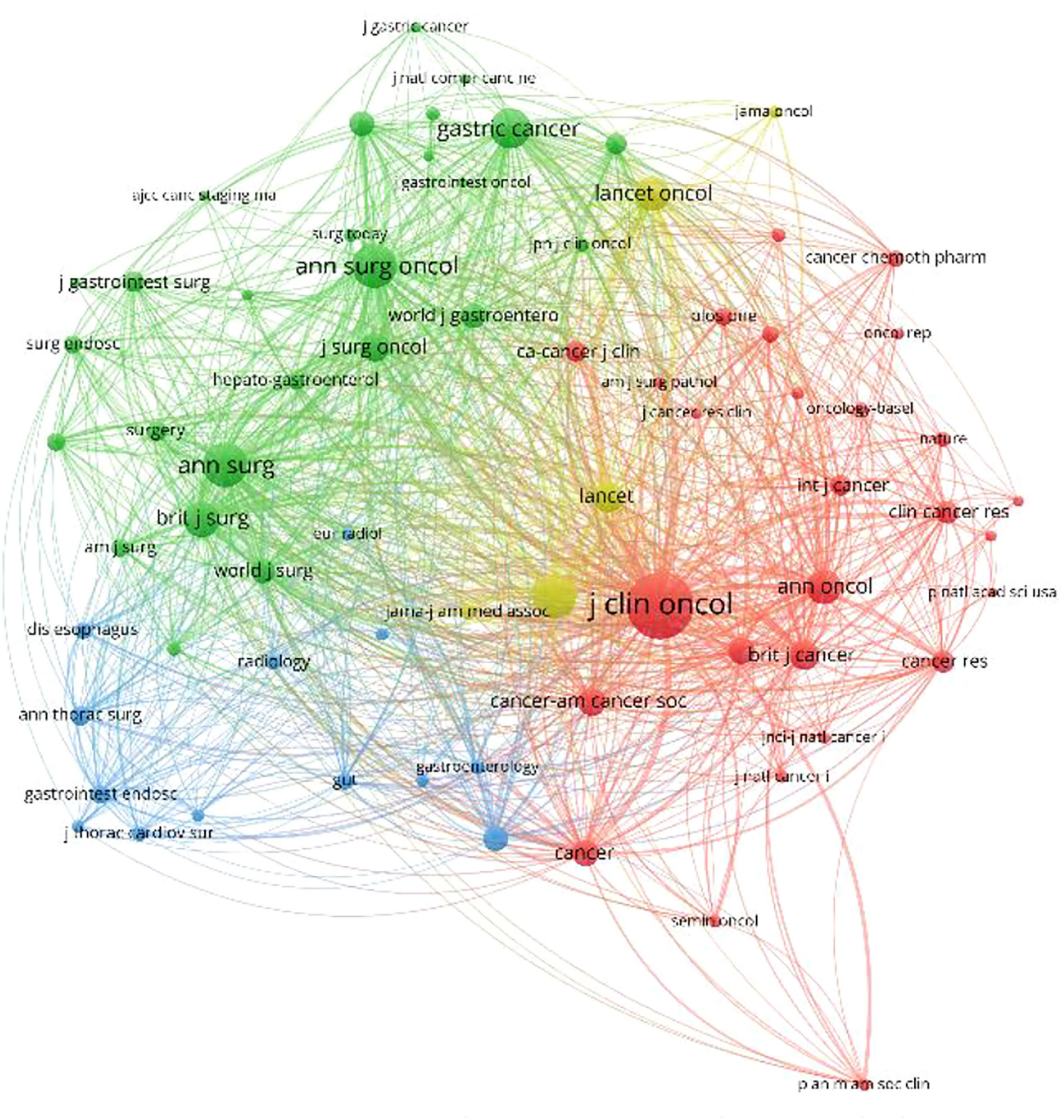
Density map of the co-cited journals, which published literature related to neoadjuvant chemotherapy for GC from 1991 to 2021.

VOSviewer software was used to draw the co-authorship and citation network among journals and authors of published studies included in this study. In **Figure 5**, each node on the chart represents a journal or author, and the size of the circle represents the number of articles published by the source journal or author. The lines connecting circles indicate citation relationship between individual journals or authors.

According to these results, the top three journals for the number of publications on neoadjuvant therapy for gastric cancer are “*Annals of Surgical Oncology*”, “*Journal of Surgical Oncology*”, and “*Gastric Cancer*”, while the “*Journal of Clinical Oncology*”, “*New England Journal of Medicine*”, and “*Annals of Surgical Oncology*” were identified as the most cited journals, as shown in **Figure 5**.

**Figure 6:**
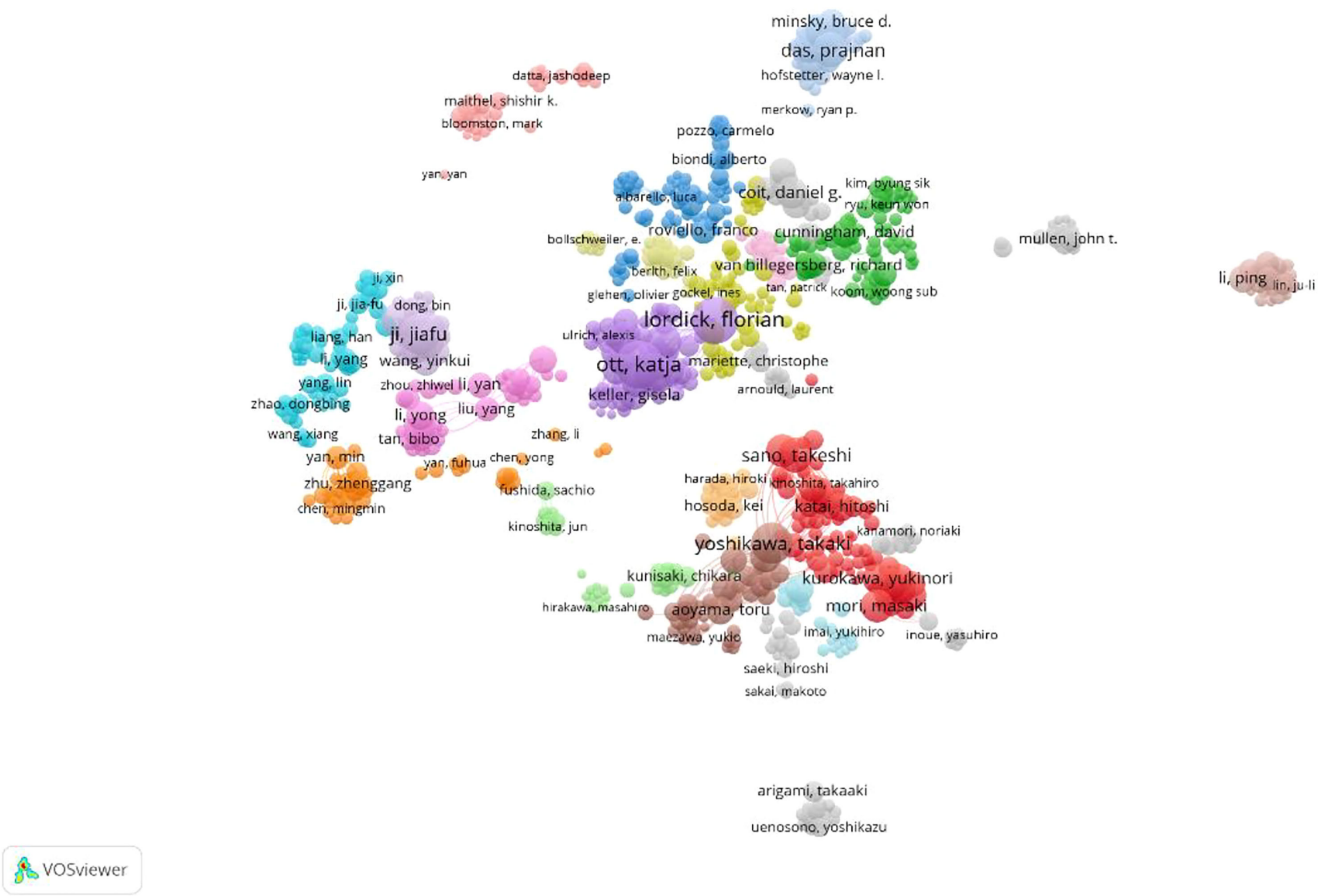
Density map of the co-cited authors, who published literature related to neoadjuvant chemotherapy for GC from 1991 to 2021.

According to the source and influence of the authors, the most published articles (n=39) related to GC neoadjuvant therapy were published by Professor Ji Jiafu from Peking University Cancer Hospital. In addition, Prof. Katja Ott and Prof. Lordick Florian of Technical University of Munich published 36 and 32 articles respectively. However, most of the high-impact authors are from Western countries. The most cited authors include Professor Cunningham David from the Royal Marsden Hospital, UK, Professor Ajani Jaffer from MD Anderson Cancer Center, USA, and Professor Ychou Marc from Montpellier Cancer Center, France. Professor Cunningham David’s publication was cited 991 times, and his 2006 tome in the New England Journal of Medicine was cited 122 times (**Figure 6**).

### 2.4 Top 10 that highly cited papers in the field of neoadjuvant therapy

The relevant information of the top 10 highly cited articles in the co-citation analysis is shown in the **Table 1**. These articles mainly focus on clinical trials of neoadjuvant therapy in GC diagnosis and treatment guidelines, Japan (22), European Society for Medical Oncology guidelines (23) and global cancer statistics completed by the International Cancer Society (24–27). Institute for Cancer Research, 2018 (28). These articles are of great significance and are the cornerstone of research in the field of neoadjuvant therapy for gastric cancer.

**Table 1:** Top 10 co-cited articles related to GC neoadjuvant chemotherapy.

Highly cited literature	Citation frequency	Journal of document source	Main content of the study
Ychou M et al. (17) (2011)	172	*J Clin Oncol*	Multicenter open-label randomized controlled phase III clinical trial
Bray F et al. (18) (2018)	159	*Ca-Cancer J Clin*	Global Cancer Statistics 2018
Al-Batran SE et al. (19) (2019)	152	*Lancet*	Multicenter open-label randomized controlled phase II/III clinical trial
Japanese Gastric Canc Assoc (20) (2017)	136	*Gastric Cancer*	Japanese Guidelines for the Treatment of GC
Van Hagen P et al. (21) (2012)	129	*New Engl J Med*	Multicenter randomized controlled phase II/III clinical trial
Cunningham D et al. (22) (2006)	122	*New Engl J Med*	Multicenter randomized controlled clinical trial
Smyth EC et al. (23) (2016)	107	*Ann Oncol*	EMSO Clinical Practice guidelines for GC
Al-Batran SE et al. (24) (2016)	107	*Lancet Oncol*	Multicenter open-label randomized controlled phase II/III clinical trial
Bang YJ et al. (25) (2012)	103	*Lancet*	Multicenter open-label randomized controlled phase III clinical trial
Ferlay J et al. (26) (2015)	98	*Int J Cancer*	Multicenter randomized controlled clinical trial

### 2.5 Research hotspots in the field of neoadjuvant therapy for gastric cancer

Statistical analysis was performed on the frequency of keywords in the published literature related to neoadjuvant therapy for gastric cancer, with a total of 768 nodes and 1494 lines, as shown in Each node in the graph represents a keyword, and the lines represent the relationship between co-occurring keywords. Also, the size of the circle indicates the frequency of the keyword.

**Figure 7:**
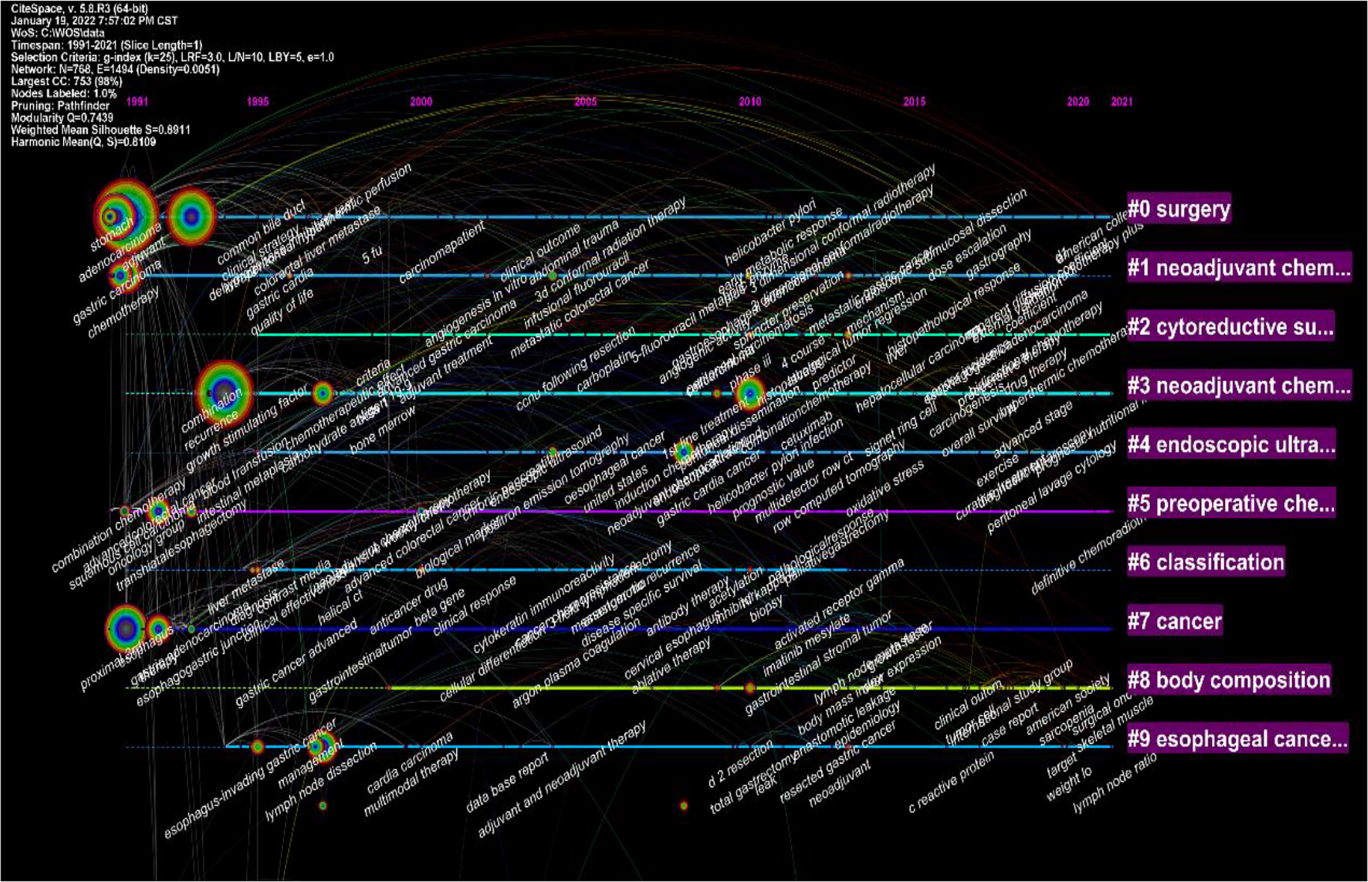
Visualization map of the timeline viewer of the studies related to neoadjuvant chemotherapy for GC from 1991 to 2021.

Noun terms were extracted from the keywords, and the logarithmic likelihood ratio algorithm was used for clustering to obtain 10 clustering items, including surgery, neoadjuvant chemotherapy, cytoreductive surgery, endoscopic ultrasonography, preoperative chemoradiotherapy, tumor, classification type, body composition analysis, esophageal cancer, etc. (**Figure 7**).

### 2.6 Research frontier of neoadjuvant therapy for GC

**Figure 8:**
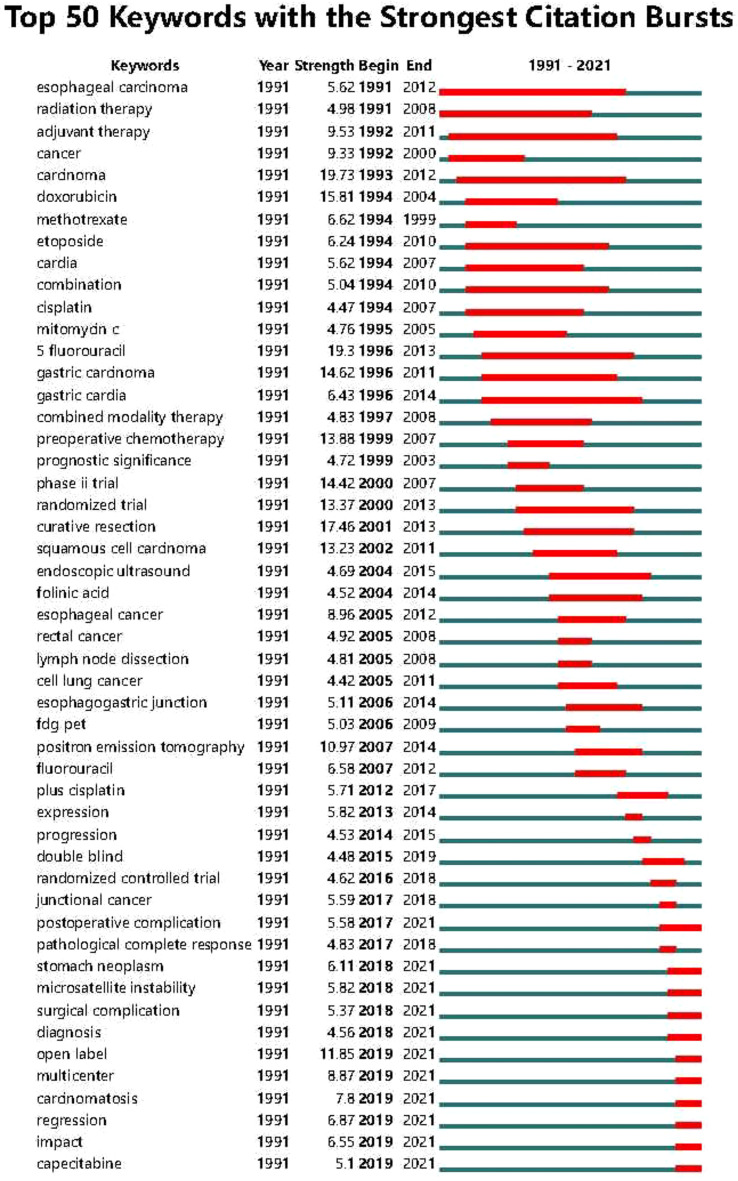
Top 50 keywords with strong citation bursts in the articles related to GC neoadjuvant chemotherapy from 1991 to 2021.

Based on the co-occurrence of keywords, a burst index and its duration were used to detect the sudden increases in the occurrence of keywords. “Postoperative complications”, “Gastric tumor”, “Microsatellite tumor”, “Microsatellite instability”, “Surgical instability,”, “Surgical complications”, “Diagnosis”, “Open-label”, “Multicenter”, “Tumor spread”, “Regression”, “Capecitabine”, and other keywords showed a upward trend. Therefore, these were the research hotspots in the field of neoadjuvant therapy for GC in the last five years. The top 50 keywords in the field of neoadjuvant therapy for GC are shown in **Figure 8**.

## 3 Discussion

### 3.1 Published papers and research strength analysis

The concept of neoadjuvant therapy for gastric cancer has gradually become popular and has become an indispensable part of gastric cancer treatment (29). Neoadjuvant chemotherapy, neoadjuvant radiotherapy, chemoradiotherapy combined with targeted immunotherapy and other related studies have continuously updated the knowledge in the field of GC treatment strategies (30, 31).

The growth trend of the annual publication volume can reflect the overall development of the research field. From 1991 to 2021, the total number of articles published on GC neoadjuvant therapy showed an upward trend, with little fluctuation. This indicates the success of neoadjuvant therapy for GC. Due to its great therapeutic potential for GC, the interest and attention of researchers in this field are still increasing. The research on neoadjuvant therapy for gastric cancer has increased rapidly in the past five years, indicating the critical significance of neoadjuvant therapy for the treatment of gastric cancer. Therefore, the focus, breadth and depth of research are constantly developing, and a large number of in-depth studies have made important progress in the application of clinical trials.

This study reviewed the literature related to GC neoadjuvant therapy and analyzed relevant information, including national and institutional journals and authors, and a collaborative network map. The results show that China, the United States and Japan carry out the highest level of research and academic activities in this field. In addition, China has the largest number of articles published in this field. Peking University, Chinese Academy of Medical Sciences and other research institutions have made contributions in the field of neoadjuvant therapy for gastric cancer. However, due to various reasons, there is still a gap between China and Western countries in the study of neoadjuvant therapy for gastric cancer, and the influence of Chinese authors and cited scholars is still lower than that of Western countries.

In addition, China has less than one-fifth of the world’s total population, but has about half of the world’s gastric cancer cases, which shows the lack and challenges of China’s gastric cancer prevention and control strategies. Relatively few patients received neoadjuvant chemotherapy. According to the relevant data report of China Gastrointestinal Cancer Surgical Alliance in 2020, the number of neoadjuvant therapy patients with locally advanced gastric cancer in 2019 was about 2 900 cases, accounting for only 13.8%. In the pathological complete rate, pCR was 11.4%, mainly in patients with neoadjuvant chemotherapy, fewer patients received neoadjuvant chemoradiotherapy (7).

### 3.2 Analysis of research hotspots and frontiers in the field of GC neoadjuvant therapy

Research hotspots can be identified by analyzing citations and keywords obtained from the literature. The rapid growth and high surge index of keywords herald the research front overview and development trend in the field of neoadjuvant therapy for gastric cancer. In the present study, a high-profile value of 0.8 was found in the top ten clusters in the GC neoadjuvant field, indicating that the clusters were homogeneous and reasonable (32).

In this study, bibliometric methods were used to analyze studies related to neoadjuvant therapy for gastric cancer, and the results were visualized using CiteSpace and VOSviewer software. Research trends in neoadjuvant therapy for gastric cancer are summarized and visualized (**Figure 7**). Highlights of the research area are explored and predictions for future research fronts are made. The study showed that among the top 10 most cited articles, most were related to clinical trials or clinical guidelines for neoadjuvant treatment of GC (**Table 1**). The analysis of the keywords revealed that recent studies have focused on the classification, diagnosis and treatment of tumors. The results also showed that “Open-label”, “Multi-center” and “Tumor spread” were the top three keywords with the highest outbreak index, indicating the research frontier of GC neoadjuvant therapy in the past five years.

### 3.3 Research deficiencies and prospects

To the best of our knowledge, this study is the first to systematically analyze publications and research trends related to GC neoadjuvant therapy in an intuitive, objective and accurate manner. The study serves as an introductory guide for clinicians and researchers in the field. However, the current research also has some unavoidable limitations or deficiencies. First, the included literature may not be exhaustive, as only the WoSCC database was searched and only articles published in English were included. Therefore, this study may not fully reflect all research in the field of neoadjuvant therapy for GC. Secondly, the included studies are limited to December 31, 2021, and some newly published or withdrawn documents may not have been updated at the time of this study due to database lag. Therefore, there may be bias or omission in the relevant literature included in this study.

Keywords such as “postoperative complications”, “gastric neoplasms”, “microsatellite instability”, “surgical complications”, “diagnosis”, “open label”, “multicenter”, “tumor spread”, “Regression” and “Capecitabine” are identified as future research frontiers in the field of neoadjuvant therapy for gastric cancer. Among them, keywords such as “stomach cancer”, “microsatellite instability”, “diagnosis”, and “tumor spread” focus on the molecular biological characteristics, pathological classification and diagnostic methods of cancer. In addition, keywords such as “postoperative complications” and “surgical complications” focused on the diagnosis, treatment, and management of complications in GC treatment, while keywords such as “open label”, “multicenter”, “regression” and “Capecitabine” focuses on new treatment methods and clinical trial design for GC neoadjuvant therapy. In order to promote the development of gastric cancer prevention and treatment strategies, future research should explore new treatment approaches on the basis of fully mining the characteristics of gastric cancer.

## 4 Conclusions

In conclusion, using bibliometric analysis, this study shows that the number of published studies related to neoadjuvant therapy for GC is gradually increasing; however, the research direction is mainly focused on the application of neoadjuvant therapy in gastric cancer to clinical trials. Multicenter randomized controlled trials and regression analysis related to neoadjuvant therapy for gastric cancer are research directions in recent years. Based on the above studies on neoadjuvant therapy for gastric cancer, the regimen and cycle of neoadjuvant chemotherapy for gastric cancer are still controversial. The “Norms for the Diagnosis and Treatment of Gastric Cancer (2018 Edition)” formulated by European ESMO and the National Health Commission of China also recommended the use of two or three drugs combined with chemotherapy regimen (platinum and fluorouracil based or combined with yew drugs), but did not recommend the use of single drugs. Although neoadjuvant chemotherapy for gastric cancer has been promoted in the past 30 years, there is currently no consensus on the selection of indications, regimen and cycle of neoadjuvant chemotherapy. Since 2001, the United States, Germany, the Netherlands, Australia, Japan, China and other countries have also conducted a large number of studies on neoadjuvant chemoradiotherapy for gastric cancer, and it has been shown that neoadjuvant chemoradiotherapy can increase pCR, reduce tumor staging, improve R0 resection rate, and make gastric cancer patients survive. However, the radiotherapy mode, dose and chemotherapy drugs of concurrent chemoradiotherapy have not been completely unified, so large-scale prospective clinical trials are urgently needed to verify the results. Neoadjuvant therapy in combination with targeted drugs may have great potential. Large phase III clinical trials are still needed to demonstrate the efficacy of combined targeted drugs. In recent years, immunocheckpoint inhibitors such as programmed death receptor 1/programmed death ligand 1 and cytotoxic T lymphocyte-associated protein 4 have been focused on tumor therapy, but their role in the treatment of many tumors, including gastric cancer, remains uncertain and there are therapeutic side effects. Although the prospect of neoadjuvant therapy for gastric cancer is promising, there is still a long way to go. At present, there is no consensus on many treatment methods in the world, which requires the joint efforts of researchers all over the world. Further research requires higher quality and high-level prospective clinical trials, so as to form more powerful, more standardized and unified clinical diagnosis and treatment guidelines, so as to bring benefits to many gastric cancer patients.

## Data availability statement

The original contributions presented in the study are included in the article/supplementary material. Further inquiries can be directed to the corresponding author.

## Author contributions

TC, First authorship: contributed to editing, revising, and approving the manuscript. DZ contributed to the writing and revision of the manuscript. YL, JG contributed to collecting clinical data. All authors contributed to the article and approved the submitted version.
